# 1,2,3-Triazole-Benzofused Molecular Conjugates as Potential Antiviral Agents against SARS-CoV-2 Virus Variants

**DOI:** 10.3390/life12091341

**Published:** 2022-08-29

**Authors:** Jehan Y. Al-Humaidi, Marwa M. Shaaban, Nadjet Rezki, Mohamed R. Aouad, Mohamed Zakaria, Mariusz Jaremko, Mohamed Hagar, Bassma H. Elwakil

**Affiliations:** 1 Department of Chemistry, College of Science, Princess Nourah bint Abdulrahman University, Riyadh 11671, Saudi Arabia; 2 Department of Pharmaceutical Chemistry, Faculty of Pharmacy, Alexandria University, Alexandria 21521, Egypt; 3 Department of Chemistry, College of Science, Taibah University, Al-Madinah Al-Munawarah 30002, Saudi Arabia; 4 Department of Chemistry, Faculty of Science, Alexandria University, Alexandria 21321, Egypt; 5 Smart-Health Initiative (SHI) and Red Sea Research Center (RSRC), Division of Biological and Environmental Sciences and Engineering (BESE), King Abdullah University of Science and Technology (KAUST), Thuwal 23955, Saudi Arabia; 6 Department of Medical Laboratory Technology, Faculty of Applied Health Sciences Technology, Pharos University in Alexandria, Alexandria 21521, Egypt

**Keywords:** anti-COVID-19, SARS-CoV-2, docking study, Omicron, 1,2,4-triazole, 1,2,3-triazole, in vitro, Vero E6

## Abstract

SARS-CoV-2 and its variants, especially the Omicron variant, remain a great threat to human health. The need to discover potent compounds that may control the SARS-CoV-2 virus pandemic and the emerged mutants is rising. A set of 1,2,3-triazole and/or 1,2,4-triazole was synthesized either from benzimidazole or isatin precursors. Molecular docking studies and in vitro enzyme activity revealed that most of the investigated compounds demonstrated promising binding scores against the SARS-CoV-2 and Omicron spike proteins, in comparison to the reference drugs. In particular, compound 9 has the highest scoring affinity against the SARS-CoV-2 and Omicron spike proteins in vitro with its IC50 reaching 75.98 nM against the Omicron spike protein and 74.51 nM against the SARS-CoV-2 spike protein. The possible interaction between the synthesized triazoles and the viral spike proteins was by the prevention of the viral entry into the host cells, which led to a reduction in viral reproduction and infection. A cytopathic inhibition assay in the human airway epithelial cell line (Vero E6) infected with SARS-CoV-2 revealed the effectiveness and safety of the synthesized compound (compound 9) (EC50 and CC50 reached 80.4 and 1028.28 µg/mL, respectively, with a selectivity index of 12.78). Moreover, the antiinflammatory effect of the tested compound may pave the way to reduce the reported SARS-CoV-2-induced hyperinflammation.

## 1. Introduction

SARS-CoV-2 (severe acute respiratory syndrome coronavirus 2) is an RNA virus (single strand) containing a spike protein gene (S) [1]. Numerous events in the SARS-CoV-2 viral replication cycle can be considered as potent targets for antiviral therapeutic development [2] starting with the viral entry, and then the membrane fusion with the host cells; next viral genome translation occurs (at the host’s endoplasmic reticulum), as does formation of the viral replication complex and double-membrane vesicles (DMVs) from the host membranes, and then the liberation of the virus particles from the cell and the replicon’s passage through the Golgi. Each of these actions entails essential viral proteins and takes place in a separate area of the host cell [3]. The spike protein (S protein) has an essential role in the viral fusion with the human angiotensin-converting enzyme 2 (ACE2), so it has become a vital therapeutic target for the development of novel anti-COVID drugs [4]. The antibacterial drug ceftazidime has been reported to be a promising spike protein inhibitor that can inhibit the membrane fusion by blocking the viral entry into the host cells [5]. After the advent of SARS-CoV-2 in December 2019, genomic monitoring revealed two lineages, several variations, and subvariants. SARS-CoV-2 variations develop through various inter- and intramolecular recombination, as well as genomic faults created throughout the genetic material [6]. The World Health Organization (WHO) and the Centers for Disease Control and Prevention (CDC) decided that the naturally occurring SARS-CoV-2 variants would be named as variants of concern (VOC) or interest (VOI) based on several factors, including the increased rates of transmission and the disease severity, recognition failures, or possible failure vaccination and the neutralizing antibodies [7,8]. Unfortunately, the emerged Delta and Omicron variants of SARS-Cov-2 showed increased transmissibility as well as pathogenicity when compared to SARS-CoV-2., These variants contain several mutations, especially in the spike protein. The Omicron variant was found to have a higher binding affinity for human ACE2 with more mutations resulting in a higher transmission potential [9]. The effectiveness of the current SARS-CoV-2 vaccinations is severely harmed by this [10]. To block viral entrance into human cells, nearly all monoclonal antibodies designed to treat SARS-CoV-2 target the spike protein. As a result, it is not unexpected that the majority of these antibodies fail to work against the Omicron form [11]. In addition, epidemiological research in South Africa revealed that the Omicron form is linked to a significantly higher risk of re-infection and a shorter doubling time [12]. The Omicron variant illness was thought to have been spread via the airborne route at a quarantine hotel in Hong Kong [13].

**Figure 1:**
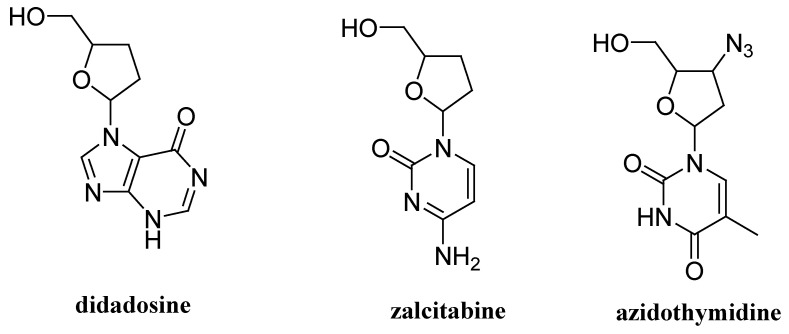
Reported reference drugs.

The triazole core is among the most significant and well-known heterocyclic scaffolds and it is found in a wide range of natural products and medicinal agents [14,15]. The broad and tunable activity of this class of triazoles has established them as pharmacologically promising scaffolds. Several commercially available drugs have been reported to possess the triazole nucleus as an important structural component [16,17]. Triazole-based derivatives have sparked great interest from research groups due to their effectiveness as therapeutic medications such as antibacterial, antifungal, antihypertensive, anticancer, and especially antiviral agents [18,19]. With regard to the antiviral activity, a triazole-based pharmacophore is likewise a highly promising agent [20] and many antiviral agents such as ribavirin analogues bear a 1,2,4-triazole moiety, proving their potential in the development of novel antiviral agents [21]. As an example, 1-benzyl-1*H*-1,2,3-triazoles-carbohydrate molecular conjugates were studied for their potential antiviral activity against HIV, and it was revealed that one compound has a greater cytotoxic effect and selectivity index (SI) than azidothymidine, zalcitabine, and didadosine (the reference drugs) (Figure 1) [22]. These compounds were chosen as leading molecules for further findings due to their theoretical profile, low cytotoxicity, and high activity. One of the most serious issues is the upsurge in synthetic drug resistance to various viruses; thus, the synthesis and formation of alternative molecular hybrids with low toxicity are critical globally and have become hot topics. The intriguing 1,2,3-triazole ring acts as a promising linker to enhance biological activity, resulting in molecular hybrid pharmacophores [23].

On the basis of these findings and in our ongoing interest in the synthesis of triazole scaffolds and their molecular hybrids with potent biological activities [24,25,26,27,28,29,30], the aim herein is the evaluation of the potential activity of a set of hybrid molecules incorporating 1,2,3- and/or 1,2,4-triazole moieties with fused heterocycles and sulfonamide tethers on SARS-CoV-2 and the new variant Omicron. Moreover, viral enzymes’ inhibition against their spike proteins in vitro Is also another goal of this research, and then the most potent molecules are further investigated by a cytopathic inhibition assay in the human airway epithelial cell line, Vero E6.

## 2. Materials and Methods

### 2.1. Chemical Synthesis

The investigated 1,2,3-triazole-benzofused hybrids were synthesized and characterized according to our previously reported work [31,32,33]. A base-assisted bis-propargylation of 2-mercaptobenzimidazole (1) with propargyl bromide afforded the targeted precursor bis-propargylated benzimidazole 2. Under optimized 1,3-dipolar cycloaddition reaction the bis-alkyne derivative (2) with ethyl azidoacetate gave benzimidazole-1,2,3-triazole hybrid 3. Hydrazinolysis, then intramolecular cyclodehydration or treatment with carbon disulfide in an ethanolic potassium hydroxide, led to the formation of 1,2,4-triazole-3-thione ring carrying benzimidazole-1,2,3-triazole hybrid 6 or 4-amino-1,2,4-triazole-3-thione-based benzimidazole-1,2,3-triazole hybrid 7, respectively.

### 2.2. Molecular Modeling

Many essential protein targets can be used for the development of novel anti-COVID medications in order to fight the global pandemic of SARS-CoV-2 and its variants. The majority of these enzyme targets play a critical role in the viral entry into the host cell (spike proteins) [34]. Thus, molecular docking studies were performed using the molecular operating environment software (MOE) to identify the binding affinity as well as the binding interactions of six target 1,2,3-triazole-sulfonamide hybrids against both SARS-CoV-2 spike protein and Omicron spike protein with the aim to discover new potent inhibitors for these principal targets [35].

#### 2.2.1. Viral Enzymes Preparation

Regarding the molecular docking calculations, X-ray crystal structures of the target enzymes SARS-CoV-2 spike protein (PDB ID: 6VW1) [36], and Omicron spike protein (PDB ID: 7wpb) [37] were obtained from the Protein Data Bank website (PDB) [38] and used as templates, followed by the protein preparation. The obtained proteins were prepared via applying the default “Structure preparation” module settings, then the MOE “Site Finder” feature was utilized to detect the binding site of the receptor.

#### 2.2.2. Database Generation and Optimization

ChemDraw program [39] was used for drawing the test compounds, then these compounds were collected in MOE software database. The database optimization was carried out through displaying hydrogen, computation of partial charges, as well as employing the default energy minimization. Triangular matcher algorithm ligand was utilized to set the ligand placement, then the top 5 non-redundant poses having the lowest binding energy of the investigated compounds were generated using the default scoring function. Docking of the optimized database was performed with the induced fitting protocol to record the best possible molecular interactions. The docking score in Kcal/mol was calculated using two scoring functions, alpha hydrogen bonding in addition to London dG forces. The obtained results were listed according to the S-scores with RMSD value < 2 Å. Results of the molecular docking can be validated using a training set of experimental ligand–protein complexes, and the accuracy of the utilized software is highly dependent on this training set [35]. To ensure a valid and reliable docking approach, the used program should be able to replicate the binding mode of a well-known reference inhibitor for the target enzyme. Although no effective anti-COVID agents are currently available, it has been reported that the antibacterial agent (ceftazidime) possesses high potential to inhibit the spike proteins and can be utilized for the development of new SARS-CoV-2 inhibitors [40]. In the trial to have positive control (reference values), the antibacterial drug ceftazidime was selected as a comparative standard for the docking study. Finally, conformers having the highest binding scores and best ligand–enzyme interactions were identified and analyzed.

### 2.3. In Vitro Antiviral Activity

SARS-CoV-2 assay kit (SARS-CoV-2 Assay Kit, Biosource, Muskego, WI, USA) and the Omicron variant Spike RBD inhibitor screening kits (B.1.1.529 BA.1, Omicron Variant Assay Kit, BPS Bioscience, San Diego, CA, USA) were used to evaluate the anti-SARS-CoV-2 activity in vitro. The suggested inhibitor screening tests were used to evaluate the binding of both spike S proteins with the ACE2 (human receptor). Different concentrations of the tested compounds were prepared, incubated for one hour at 37 °C with spike S protein, then the optical density (OD) was monitored using an ELISA reader (at 450 nm) [41].

### 2.4. Antiviral Activity in Infected Cell Line Using Cytopathic (CPE) Inhibition Assay

Vero E6 cell line was used for a CPE inhibition assay through drug susceptibility testing. Cells were grown in titer plates inoculated with Dulbecco’s modified eagle medium (DMEM) (Grand Island, NY, USA) supplemented with 0.1% antibiotic/antimycotic solution and 10% fetal bovine serum. The present investigation was conducted to evaluate the antiviral activity and cytotoxicity assays of the tested compounds using the cytopathic (CPE) inhibition effect according to Donalisio et al. [42]. The development of cytopathic effect was examined by light microscope. The optical density of each inoculated well was quantified spectrophotometrically at 570/630 nm. The percentage of antiviral activities of the tested compounds were calculated according to Pauwels et al. [43] using the following equation

(1) $$\mathrm{Antiviral}\;\mathrm{activity}=\frac{\mathrm{mean}\;\mathrm{optical}\;\mathrm{density}\;\mathrm{of}\;\mathrm{cell}\;\mathrm{controls}-\mathrm{mean}\;\mathrm{optical}\;\mathrm{density}\;\mathrm{of}\;\mathrm{virus}\;\mathrm{controls}\;}{\mathrm{mean}\;\mathrm{optical}\;\mathrm{density}\;\mathrm{of}\;\mathrm{virus}\;\mathrm{controls}}\times 100$$

Based on these results, the 50% CPE inhibitory dose (EC50) and the 50% cytotoxic concentrations (CC50) were calculated.

### 2.5. Evaluation of Proinflammatory Cytokine Production

The proinflammatory markers (IL-1β and IL-6) were quantified in the normal, infected viral cells (without treatment) and infected viral cells treated with the most promising compound according to the ELISA kit method (Immunotag™ ELISA Kits) at 450 nm [44].

## 3. Results and Discussion

**Scheme 1:**
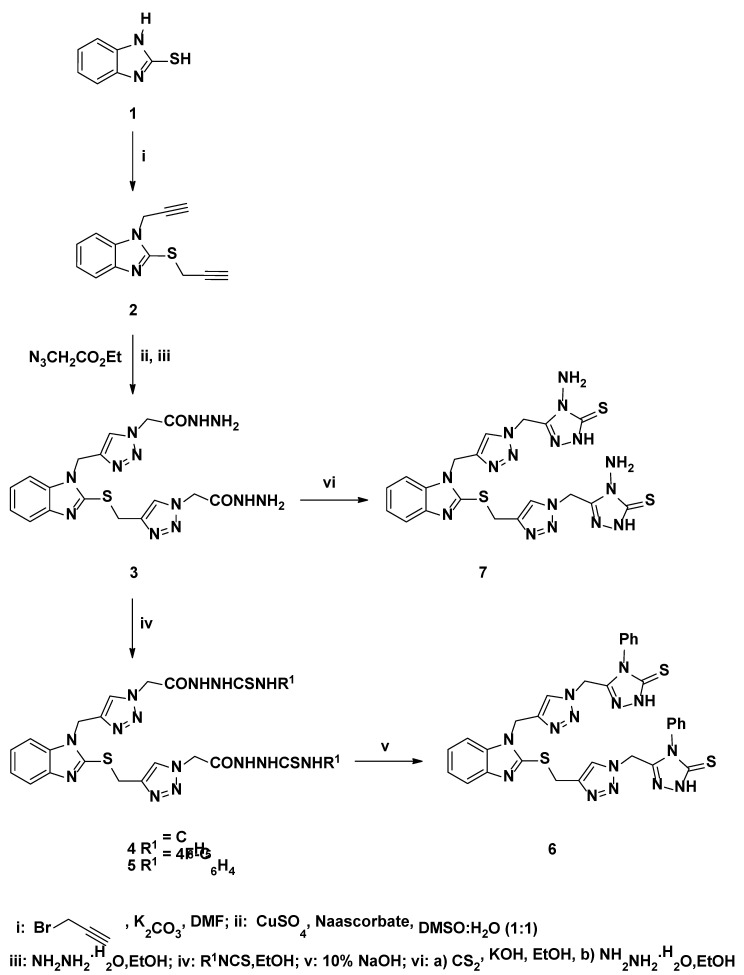
Synthesis of benzimidazole-1,2,3-triazole hybrids carrying 1,2,4-triazole 6 and 7.

The tested benzimidazole-1,2,3-triazole hybrids 6 and 7 were prepared via multistep synthesis (Scheme 1) according to our previously reported procedures [31]. The synthesis first required base-assisted bis-propargylation of 2-mercaptobenzimidazole (1) with propargyl bromide in the presence of potassium carbonate as a catalyst to afford the targeted precursor bis-propargylated benzimidazole 2. Under optimized Cu(I) conditions (CuSO_4_, Na-ascorbate), the click 1,3-dipolar cycloaddition reaction of the bis-alkyne derivative 2 with ethyl azidoacetate furnished the formation of the desired benzimidazole-1,2,3-triazole hybrid with ester tether. Hydrazinolysis of the later ester, in refluxing ethanol for 4 hr, yielded the acid hydrazide 3, which upon condensation with the appropriate aryl isothiocyanate derivatives, gave the corresponding acid thiosemicarbazide derivatives carrying benzimidazole-1,2,3-triazole conjugates 4 and 5. Intramolecular cyclodehydration of the phenyl acid thiosemicarbazide derivative 4 in refluxing 10% of the aqueous sodium hydroxide solution led to the formation of the corresponding 1,2,4-triazole-3-thione ring carrying benzimidazole-1,2,3-triazole hybrid 6. On the other hand, the treatment of the acid hydrazide 3 with carbon disulfide, in an ethanolic potassium hydroxide solution, afforded the xanthate salt, which underwent an intramolecular cyclization in the presence of hydrazine hydrate to afford the desired 4-amino-1,2,4-triazole-3-thione based benzimidazole-1,2,3-triazole hybrid 7. Full characterization of the newly designed 1,2,3-triazole hybrids has been reported in detail in our previously reported work [31] (Scheme 1).

**Scheme 2:**
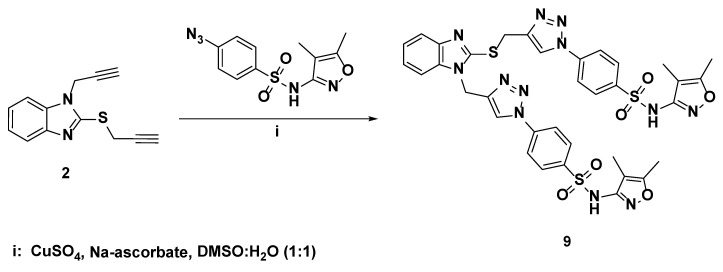
Synthesis of benzimidazole-1,2,3-triazole hybrid carrying sulfaoxazole 9.

Moreover, the synthesis of the specific targeting of bis-benzimidazole-1,2,3-triazole molecular conjugate containing sulfaoxazole core 9 was investigated via the 1,3-dipolar cycloaddition reaction of the bis-S,N-propargylated benzimidazole 2 with 4-azido-N-(4,5-dimethylisoxazol-3-yl)benzenesulfonamide (8) using the same Cu(I)-catalyzed click conditions reported in our previous work [32] (Scheme 2).

**Scheme 3:**
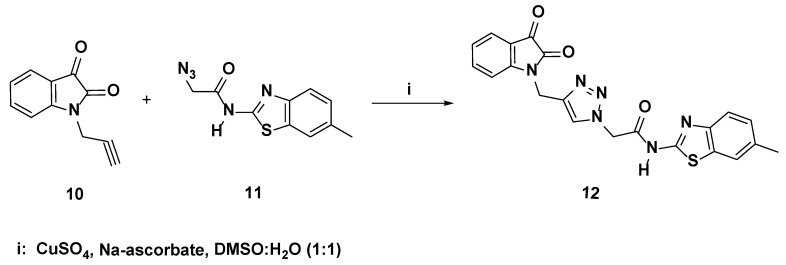
Synthesis of isatin-1,2,3-triazole hybrid carrying benzothiazole moiety 12.

In the present work, we also reproduced the synthesis of isatin-1,2,3-triazole molecular conjugate with benzotriazole tether 12 according to our previous work [33]. The synthesis required the click ligation of isatin-based alkyne 10 with 2-azido-N-(6-methylbenzo[d]thiazol-2-yl)acetamide 11 in a mixture of DMSO:H_2_O (1:1, *v*/*v*) as a solvent, at room temperature for 12 h, to afford compound 10 in an 86% yield (Scheme 3).

### 3.1. Molecular Docking Calculations

**Figure 2:**
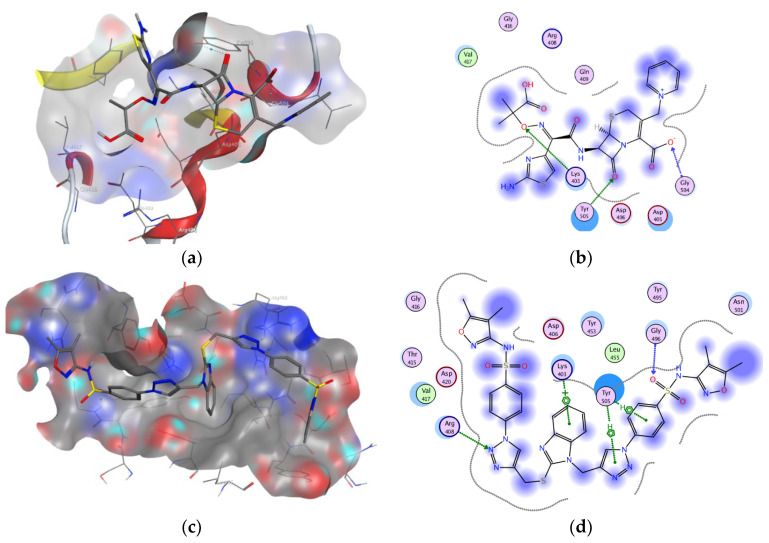
(**a**) Three-dimensional binding mode of ceftazidime, (**b**) 2D binding mode of ceftazidime, (**c**) 3D binding mode of compound **9**, (**d**) 2D binding mode of compound **9** within SARS-CoV-2 spike protein binding site.

**Figure 3:**
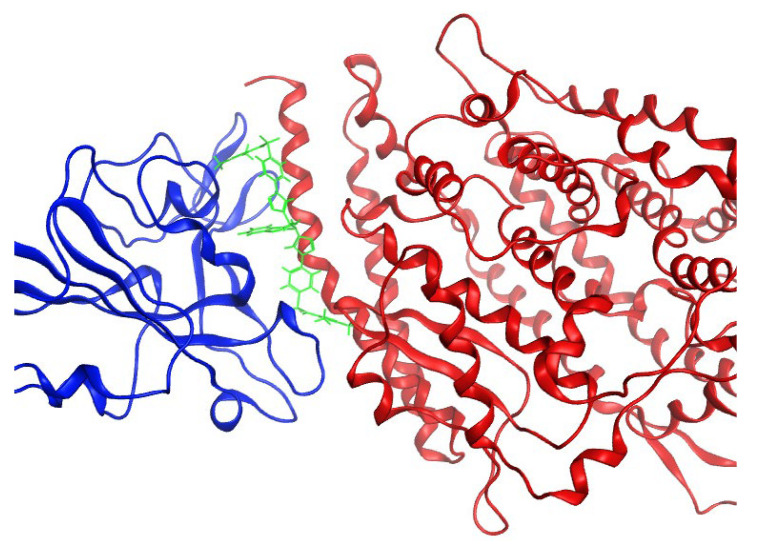
Three-dimensional binding mode of compound 9 (green) at the binding interface between the SARS-CoV-2 S-RBD (red) and human ACE2 (blue).

**Figure 4:**
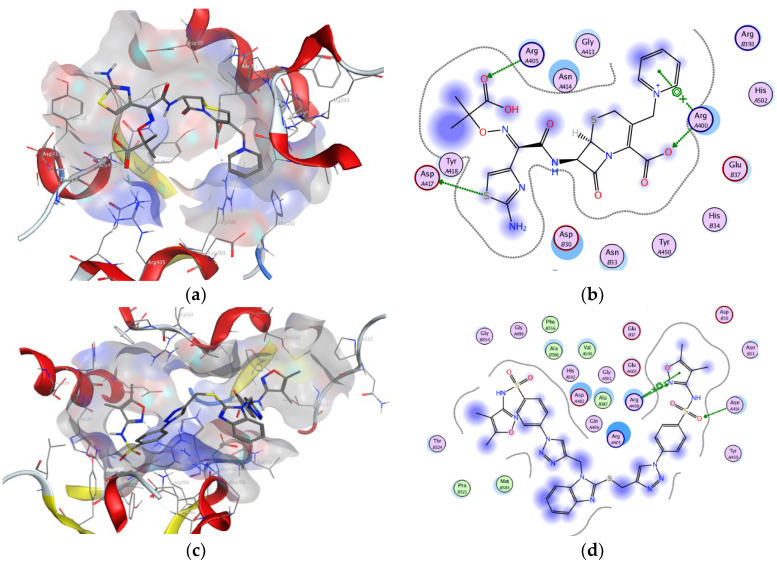
(**a**) Three-dimensional binding mode of ceftazidime, (**b**) 2D binding mode of ceftazidime, (**c**) 3D binding mode of compound **9**, (**d**) 2D binding mode of compound **9** within Omicron spike binding site.

**Figure 5:**
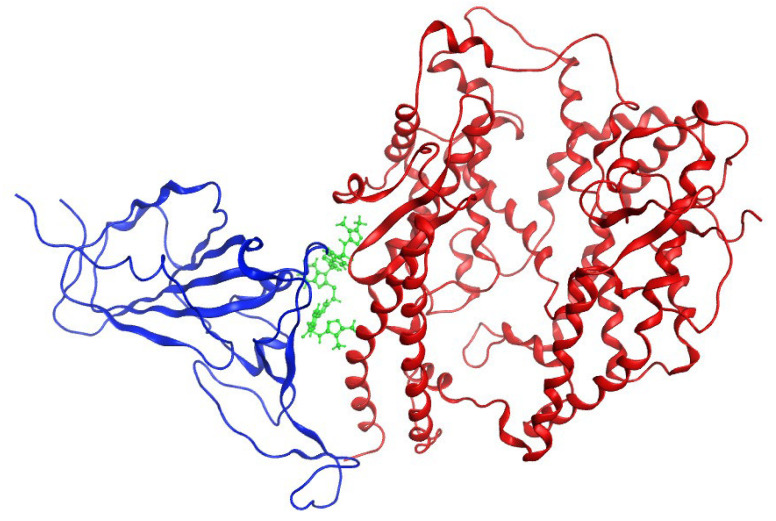
Three-dimensional binding mode of compound 9 (green) at the binding interface between the Omicron S-RBD (red) and human ACE2 (blue).

The predicted docking scores in addition to the binding interactions of the investigated compounds toward the target enzymes (SARS-CoV-2 spike protein S1 and the Omicron spike) are listed in Table 1 and Table 2. In addition, the 2D and 3D representations of interaction between the inspected compounds and the enzyme active site are presented in Figure 2, Figure 3, Figure 4 and Figure 5.

#### 3.1.1. SARS-CoV-2 Spike Protein

The data are tabulated in Table 1 and reveal that compounds 4, 5, 6, and 9 demonstrated higher binding scores than the known reference inhibitor ceftazidime (−6.36 Kcal/mol) against the SARS-CoV-2 spike. In particular, compound 9 (Figure 2) exhibited the best binding score (−7.27 Kcal/mol) among all test compounds. It is worth mentioning that most of the investigated compounds displayed a strong pattern of interaction with the enzyme similar to that exhibited by ceftazidime, which indicates a valid reliable docking approach. It was also observed that test compounds can dock deeply within the receptor-binding domain (RBD) of the spike protein displaying hydrogen bond interactions with the key amino acids LYS403 and ARG408, resulting in destabilization or even prevention of the spike protein–ACE2 interaction. Compound 9, possessing the highest binding score, was chosen for further testing to determine its binding affinity with the spike RBD or human ACE2. The docking of compound 9 at the interface between the spike RBD and human ACE2 (PDB ID: 6VW1) (Figure 3) revealed strong and stable interactions with the spike RBD, while exhibiting no binding interactions with ACE2.

Murugavel et al. [45] studied the molecular docking of triazole derivatives as inhibitors of the SARS-CoV-2 spike protein, and it was revealed that the binding energy ranged from −8.8 to −8.9 Kcal/mol with several interactions, namely, hydrogen bonds, electrostatic, and hydrophobic, with the key amino acids (ARG355, ARG466, GLU465, LYS462, GLU516, TRP353, TYR396, LEU518, and PHE464) that validate the stability and the potential medicinal activity of the tested triazole derivatives.

#### 3.1.2. Omicron Spike Protein

According to the docking results listed in Table 2, all tested compounds except compound 12 showed remarkable binding scores ranging from −7.90 to −8.66 Kcal/mol, which is higher than the reference ceftazidime (docking score −6.59 Kcal/mol) (Figure 4). Docking results revealed that all investigated molecules fitted well at the interface of the Omicron spike in complex with human ACE2; in addition, they can form different types of interactions with S-RBD such as hydrogen bonding and hydrophobic interactions, leading to destabilization or prevention of the spike protein–ACE2 interaction. It was also found that compound 9 (having the best binding score) showed a strong pattern of interaction with S-RBD (Figure 5). By contrast, this compound showed no binding interactions with ACE2.

### 3.2. In Vitro Enzyme Activity

**Figure 6:**
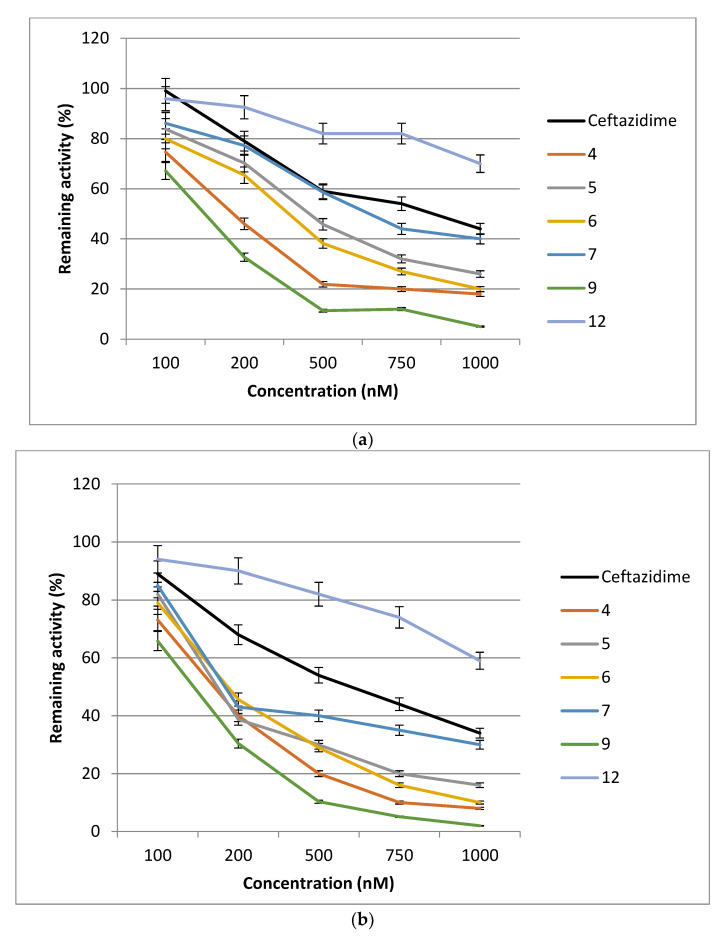
The remaining viral binding activity of the tested compounds against (**a**) SARS-CoV-2 spike protein (**b**) Omicron spike protein.

Data proved the promising activity of the tested compound 9 with its IC50 reaching 75.98 nM against the Omicron spike protein and 74.51 nM against the SARS-CoV-2 spike protein, while the lowest activity was recorded with compound 12 (Figure 6 and Tables S1 and S2). SARS-CoV-2 was more resistant (with a higher IC50) to the tested compounds; hence, it was chosen for further analyses in the human cell line.

In the present study our main goal was to inhibit the viral entry into the host cell; hence, we targeted the SARS-CoV-2 and Omicron spike proteins. However, other studies have focused on other viral enzymes such as the 3C-like protease (3CLpro), the essential protease enzyme that cleaves the replicase polyproteins during viral replication. Karypidou et al. [46] studied the effect of novel 1,2,3-triazoles as potent anti-SARS-CoV-2 agents by targeting 3CLpro. Benzotriazoles had the highest inhibition activity with an IC50 equal to 0.2 μM due to the interaction with Cys145, Ser144, and Gly143. Moreover, five 1,2,3-triazole compounds showed moderate activity against HcoV-229E with a 50-fold lower EC50 when compared to the reference Urtica dioica agglutinin (UDA) (EC50 = 0.2 μM).

### 3.3. Cytopathic (CPE) Inhibition Assay

This is the first work to study some 1,2,3-Triazole-benzofused molecular conjugates as potent anti-SARS-CoV-2 compounds by using CPE inhibition assay. The tested compounds were added in serially diluted concentrations and compared to untreated infected cells, and then the EC50 and CC50 values were recorded (Table 3). Our studies revealed that SARS-CoV-2 was effectively inhibited by compound **9** with a high selectivity index (SI), which proved the drug’s safety and compatibility for future application and assessment in an animal model. Because of their lower free energy, triazoles produced the most stable compound with the spike protein. These interactions are thought to cause conformational changes in the viral structural proteins, preventing virus entry into host cells, and hence reducing virus reproduction and infection.

Seliem et al. [47] designed and synthesized some quinolone–triazole conjugates against SARS-CoV-2. It was revealed that 4-((1-(2-chlorophenyl)-1H-1,2,3-triazol-4-yl)methoxy)-6-fluoro-2-(trifluoromethyl)quinoline (10 g) and 6-fluoro-4-(2-(1-(4-methoxyphenyl)-1H-1,2,3-triazol-4-yl)ethoxy)-2-(trifluoromethyl)quinoline (12c) have a high antiviral activity with a high selectivity index (SI) against SARS-CoV-2 in comparison to the reference drugs. They explained that the fluorine atoms in the tested compounds have the major role in the observed antiviral activity.

### 3.4. Evaluation of Proinflammatory Cytokine Production

**Figure 7:**
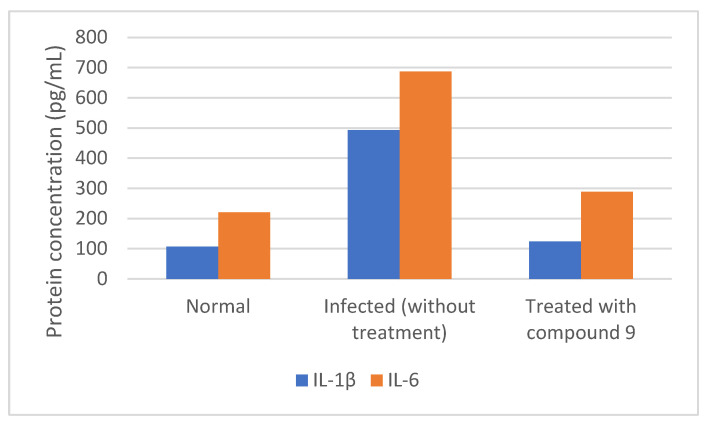
Antiinflammatory effect of compound **9**.

According to surveys, patients with severe COVID-19 may experience a hyperinflammatory condition. Therefore, it is critical to discover new strategies for reducing excessive inflammation in order to lower the death rates. According to our results, all of the inflammatory cytokines’ (IL-1β and IL-6) protein expression levels were noticeably reduced when compound 9 was used (Figure 7). These findings showed that compound 9 has antiviral activity and reduces SARS-CoV-2-induced hyperinflammation.

## 4. Conclusions

Currently, a number of simulation studies are being carried out to discover some potential drugs to combat SARS-CoV-2. With the use of these investigations, it is possible to discover various treatment options with known interactions with the targeted proteins, biosynthetic pathway, and bioactivity scores, and the compatibility with some known drugs. Molecular docking was used in the current investigation to predict the binding characteristics and affinities of some 1,2,3-triazole-sulfonamide hybrids against the targets’ enzymes, namely, the SARS-CoV-2 spike and the Omicron spike proteins. The results of the docking analyses revealed that the majority of the compounds under investigation showed promising binding scores with the active site of the target enzymes. In particular, compound 9 exhibited the best scoring results with many types of interaction with the target enzymes. In vitro enzyme inhibition and cytopathic inhibition assays in human Vero E6 cell line tests proved the potential antiviral effect of the synthesized 1,2,3-triazole-sulfonamide hybrid as an effective and safe drug against SARS-CoV-2 and Omicron infections.

## Figures and Tables

**Table 1:** Calculated docking scores (in kcal/mol) and binding features for the drug candidates against SARS-CoV-2 spike protein.

Compound Code	Docking Score (kcal/mol)	Binding Features (Hydrogen Bond)
Ceftazidime	−6.36	LYS403, GLY504, TYR505
**4**	−6.52	LYS403, ARG408, THR415
**5**	−6.73	ARG408, THR415, GLN493
**6**	−6.84	LYS403, ASP406, THR415, GLY496
**7**	−6.24	LYS403, ASP406, ARG408
**9**	−7.27	ARG408, GLY496
**12**	−5.27	LYS403, ARG408

**Table 2:** Calculated docking scores (in kcal/mol) and binding features for the drug candidates against Omicron spike protein.

Compound Code	Docking Score (kcal/mol)	Binding Features (Hydrogen Bond)
Ceftazidime	−6.59	ARG400, ARG405, ASP417
**4**	−8.33	ARG400, ARG405, ASN414
**5**	−8.02	ASN414, HIS502
**6**	−8.04	ARG400, ASP402
**7**	−7.90	ARG400, ASP402
**9**	−8.66	ARG400, ASN414
**12**	−6.14	ASN414

**Table 3:** Antiviral activity of the synthesized 1,2,3-triazole-benzofused conjugates.

Tested Compounds	CC50 (µg/mL)	EC50 (µg/mL)	Selectivity Index (SI)
Ceftazidime	1045.53	85.07	12.29
**4**	1899.87	174.3	10.90
**5**	2035.65	230.8	8.82
**6**	1065.51	155.05	6.87
**7**	1530.5	306.1	5.0
**9**	1028.28	80.4	12.78
**12**	331.90	381.5	0.87

## Data Availability

The data presented in this study are available on request from the corresponding authors.

## Supplementary Materials

The following supporting information can be downloaded at: https://www.mdpi.com/article/10.3390/life12091341/s1, Table S1. In vitro assay of the remaining activity % of omicron spike proteins after using the synthesized 1,2,3-Triazole-benzofused compounds. Table S2. In vitro assay of the remaining activity % of SARS-CoV-2 spike proteins after using the synthesized 1,2,3-Triazole-benzofused compounds.
