# Supplementary material for: 1,2,3-Triazole-Benzofused Molecular Conjugates as Potential Antiviral Agents against SARS-CoV-2 Virus Variants
Source: Life (Basel). 2022 Aug 29;12(9):1341. doi: 10.3390/life12091341 (PMC9502539; doi:10.3390/life12091341)
Supplement: Supplementary file 1 [file life-12-01341-s001.zip › life-1815689-supplementary.pdf]

1,2,3-Triazole-benzofused molecular conjugates as potential antiviral agents against SARS-CoV-2 virus variants

Supplementray data

Table S1. In vitro assay of the remaining activity % of omicron spike proteins after using the synthesized 1,2,3-Triazole-benzofused compounds

Tested compounds	Tested concentrations (nM)				
	100	200	500	750	1000
Ceftazidime	89	68	54	44	34
**4**	73.1	40	20	10	8
**5**	82	38.7	30	20	16
**6**	79	45.6	29	16	10
**7**	85	43	40	35	30
**9**	65.8	30.4	10.3	5.1	2
**12**	94	90	82	74	59

Table S2. In vitro assay of the remaining activity % of SARS-CoV-2 spike proteins after using the synthesized 1,2,3-Triazole-benzofused compounds

Tested compounds	Tested concentrations (nM)				
	100	200	500	750	1000
Ceftazidime	99	79	59	55.1	45.2
**4**	74.6	46	21.9	20	18
**5**	83.8	70.2	45.8	32	26
**6**	79.9	65.4	38.2	27	20
**7**	86.1	77.2	58.6	44	40
**9**	67.1	32.7	11.4	12	5
**12**	95.9	92.5	82	82	70
